# New Challenges and Perspectives in Polycystic Ovary Syndrome

**DOI:** 10.3390/jcm14248867

**Published:** 2025-12-15

**Authors:** Jim Parker, Vanessa Hitch

**Affiliations:** 1 School of Medicine, University of Wollongong, Wollongong 2522, Australia; 2 College of Complementary Medicine, Sydney 2010, Australia; vanessa@seekvitality.com

## 1. Introduction

Medical knowledge and practice continue to evolve at a rapid pace. This Special Issue explores new challenges and perspectives in research and reviews related to polycystic ovary syndrome (PCOS). Along with other non-communicable diseases (NCDs)—such as obesity, type 2 diabetes mellitus (T2DM), metabolic dysfunction-associated steatotic liver disease (MASLD), cardiovascular disease (CVD), and mental health disorders [1,2]—PCOS is now recognized as an *evolutionary mismatch disorder* that manifests when survival traits adapted to ancestral environments become maladaptive in the modern world [3,4,5]. The emergence of this new paradigm results from the application of systems theory to human health and allows for a more holistic view of PCOS [3,6,7]. This is reflected in the International Guidelines for PCOS [8], which recommend a “lifestyle-first” approach to management, and an appreciation of the complex interaction between neuroendocrine and the immune and metabolic homeostatic networks that regulate human physiology [9,10].

The patient is no longer a passive recipient in their relationships with health professionals, but rather an active participant in medical care. Adopting an evolutionary perspective provides a framework for promoting healthy lifestyles and preventative interventions and forms a central component of a new insulin-centric model proposed by Parker et al. for the assessment and management of PCOS (contribution 4). The new discipline of *narrative medicine* represents a focus on the needs of the patient and their active involvement in healthcare [11]. Narrative medicine emphasizes listening to patients’ stories to foster empathy, meaning, and personalized holistic care [12]. Framing patient stories in an evolutionary context allows struggles involving lifestyle risk factors such as diet, exercise, sleep, and stress to be discussed in their broader evolutionary context. This can create empathy through shared human history and helps patients feel less “at fault” and more connected to a community, providing a narrative reframing for behavior change. Evolutionary explanations provide biological meaning, while narrative frameworks in medicine ensures those explanations are personalized and emotionally relevant. Introducing an evolutionary perspective may provide a welcome balance in this new era of “triadic” consultation, whereby the clinician, patient, and artificial intelligence (AI) shape clinical encounters together [13].

**Figure 1:**
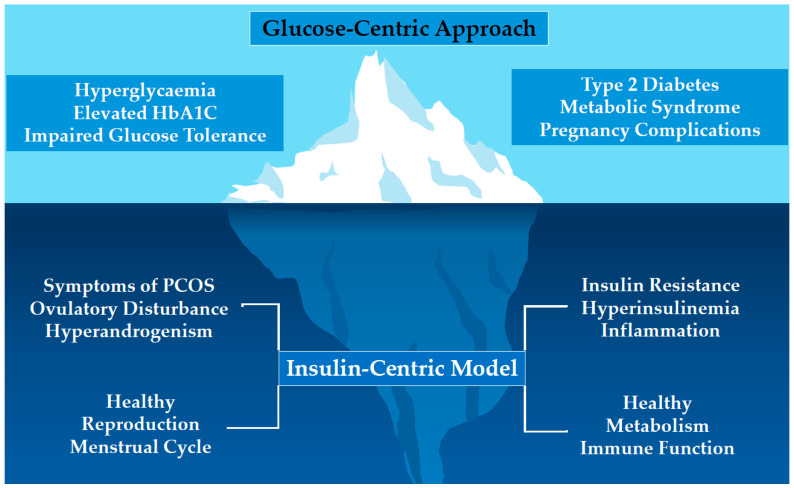
The observed features of the glucose-centric approach represent the late stages of the effects of insulin resistance or the ‘’tip of the iceberg”. HbA1C = Hemoglobin A1C. Adopted from Parker et al.

In addition to the emerging evolutionary paradigm, PCOS is now recognized as a multisystem metabolic and endocrine disorder, rather than a primary gynecological condition, as the name suggests. This is reflected in the 2023 International Guidelines that emphasize the broad metabolic, reproductive, and psychological aspects of PCOS [8], in a recent analysis of the Global Burden of Disease data that highlights the environmental and behavioral drivers [14], and in the current appeal to change the name, having reached a global consensus [15]. Insulin resistance (IR) and hyperinsulinemia are central mechanisms in metabolic dysfunction and form core components of the pathophysiology of PCOS [16,17]. Genetic polymorphisms in the insulin receptor genes and variants linked to insulin signaling and glucose metabolism (INSR, IRS1/2, PPAR-Ɣ), reducing insulin sensitivity and worsening metabolic features of PCOS [18]. As a result, women with PCOS have a 27% reduction in insulin sensitivity, independent of obesity [19]. Early diagnosis of IR would facilitate timely implementation of effective treatment strategies (see contributions 4 and 6) and potentially limit the progression of metabolic-related mismatch diseases (Figure 1).

**Figure 2:**
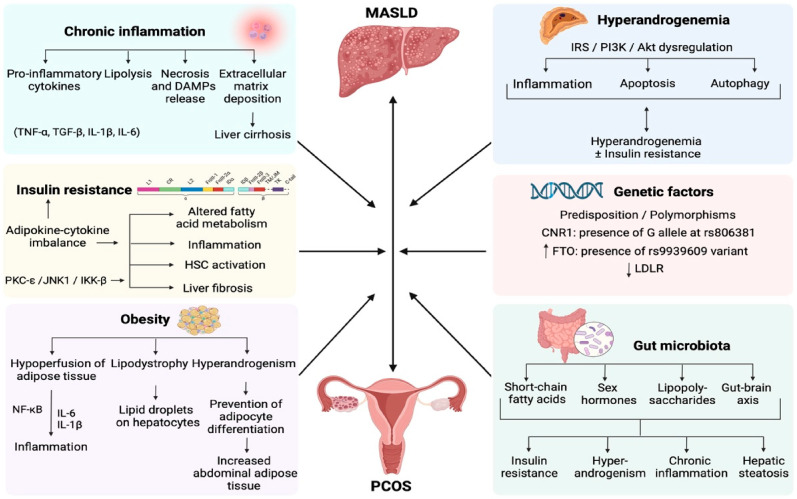
Schematic presentation of the pathophysiological mechanisms correlating MASLD with PCOS. Insulin resistance disturbs fatty acid and hepatic lipid metabolism, leading to a chronic low-grade inflammatory state and fibrosis, via the activation of HSCs. Obesity is associated with inflammation, lipodystrophy, and elevated androgen levels, while hyperandrogenemia is correlated with inflammation, apoptosis, and autophagy. Genetic factors, including predisposition and polymorphisms, play a crucial role in the development of MASLD and PCOS, while gut microbiota dysbiosis is linked with insulin resistance, hyperandrogenemia, inflammation, and steatosis. Chronic inflammation associated both with MASLD and PCOS results in a necro-inflammatory pro-fibrinogenic hepatic environment, predisposing to liver cirrhosis. Created with BioRender.com. Abbreviations include DAMPs: damage-associated molecular patterns; IKK-β: kappa-B kinase subunit beta; HSC: hepatic stellate cells; IL-1β: interleukin 1β; IL-6: interleukin 6; IRS-PI3K-Akt: Insulin receptor substance-phosphoinositide-3-kinase-Ak strain transforming; JNK1: c-Jun N-terminal kinases 1; MASLD: metabolic dysfunction-associated steatotic liver disease; NF-κB: nuclear factor kappa-light-chain-enhancer of activated B cells; PCOS: polycystic ovary syndrome; PKC-ε: protein kinase C epsilon; TNF-α: tumor necrosis factor-α; and TGF-β: transforming growth factor-β. Adopted from Arvanitakis et al.

Taken from an evolutionary perspective, reduced insulin sensitivity is an adaptive protective mechanism that increases survival chances during periods of increased environmental (starvation, infection) or physiological demand (pregnancy, stress) [3]. Modern lifestyle and environmental exposures result in maladaptive responses that increase the risk of other metabolic-related mismatch disorders [20,21]. As a result, lifestyle and medical interventions are effective at preventing and treating IR and related metabolic disease, including PCOS [8]. A second review in this Special Issue addresses gaps in understanding the role of IR in the pathophysiology of metabolic dysfunction in PCOS. Arvanitakis et al. (contribution 5) discuss the complex interplay between IR and other pathophysiological processes (e.g., chronic inflammation, hormonal imbalance, gut dysbiosis) in MASLD. This review highlights common pathophysiological mechanisms linking MASLD and PCOS (Figure 2).

Immune dysregulation is also a central component of the pathophysiology of PCOS and forms part of the homeostatic network involving metabolism, hormone imbalance, and inflammation [10]. Inflammation is an evolutionarily conserved physiological process designed to be short-lived in order to contain pathogens and respond to trauma [22]. In 2012, Tremellen and Pierce proposed a novel framework—*dysbiosis of gut microbiota*—to describe the pathogenesis of PCOS [23]. The authors hypothesized that poor-quality diet induced gut-microbial imbalance resulting in the disruption of the gastrointestinal mucosal barrier, transmigration of lipopolysaccharide, activation of Toll-like receptors on submucosal macrophages, and release of inflammatory cytokines. This process initiates chronic systemic inflammation (CSI) and contributes to IR and hormonal imbalance in PCOS. Many proof-of-concept studies support this hypothesis [24] and many additional mechanisms have now been described (e.g., altered bile acid composition, choline, lactate, and trimethylamine N-oxide), in addition to lipopolysaccharide-induced inflammation [25]. Similar mechanisms have been identified in other NCDs associated with PCOS [26].

Chronic low-grade systemic inflammation or *metaflammation*, caused by poor-quality diet, nutritional excess, gut-microbial imbalance, stress, and other environmental factors, interacts with metabolic and hormonal signaling pathways and contributes to symptoms and disease progression in PCOS [10]. Although the *common mucosal system* was described over 50 years ago, it has only recently been recognized to have a role in PCOS [27]. The common mucosal immune system links the mucosal surfaces of the body (with a surface area of 400 m^2^) via circulating immune cells [28]. It is now evident that CSI can be initiated at any mucosal surface (e.g., gut, respiratory, reproductive, skin) by a variety of environmental substances (microparticulate air pollution, microplastics, endocrine-disrupting chemicals), in addition to dietary components [27]. Women with PCOS also appear to have an evolutionarily conserved *proinflammatory design* that was protective in an ancestral environment but becomes maladaptive in the modern world [10]. This collection of papers includes a systematic review by Alenezi et al. (contribution 6) on the impact of diet-induced weight loss on inflammatory status and hyperandrogenism in PCOS.

Many diagnostic and prognostic biomarkers have been investigated for their potential role in PCOS [29]. These include hormonal (LH/FSH ratio, androgens, AMH, SHBG), metabolic (HOMA-IR, OGTT), inflammatory (CRP, IL-6, TNF-α), and genetic (micro-ribonucleic acid, differentially expressed genes) biomarkers. Reliable biomarkers may contribute to early diagnosis, risk stratification, and personalized management. In general, they are best used in combination with clinical criteria and other investigations, rather than as standalone diagnostic tools. One exception is AMH, which is now included in the International Guidelines as a component of the diagnostic criteria in adults—but not adolescents—with PCOS [8]. Salivary biomarkers provide an accessible non-invasive tool for investigating PCOS. One previous study has explored the role of salivary inflammatory markers and testosterone in gingival health in adolescents with PCOS [30]. This Special Issue includes the first pilot study on the diagnostic potential of *salivary biomarkers* for inflammation and hyperandrogenism (contribution 2).

Mental health concerns are increasingly recognized as significant features of PCOS, with consistent evidence showing higher rates of depression, anxiety, and psychological distress relative to women without PCOS [31]. A 2023 systematic review reported that women with PCOS have a more than 2.5-fold higher risk of depression compared with healthy controls [32]. While physical manifestations such as hirsutism, weight changes, and fertility difficulties can add to emotional strain, research indicates that the psychological burden is closely driven by heightened perceived stress, reduced self-efficacy, and the cumulative pressures of living with a chronic multisystem condition. This pattern was confirmed in the study by Marschalek et al. (contribution 3), where psychological distress correlated closely with perceived stress and helplessness rather than hormonal or metabolic measures. Chronic stress may also heighten neuroendocrine and metabolic dysregulation via altered HPA-axis and adrenal steroid responses [33]. Collectively, these findings support integrated mental health screening and stress-focused interventions in PCOS care.

*Evolutionary psychology* holds the potential to improve our understanding of the link between physical and psychological wellbeing [34]. The higher levels of psychological symptoms experienced by women with PCOS may also have evolutionary origins. Evolutionary psychology assumes that the human mind has evolved to solve problems relevant to human survival and reproduction by means of psychological adaptations. These adaptations are fine-tuned to ancestral living but may negatively influence health in modern environments—such as when highly processed, ultra-palatable, addictive foods are easily available [35]. Reliable mental health assessment tools are needed to help identify women who require support with emotional regulation and coping strategies (contribution 3).

A wide range of therapeutic options are available for the treatment of PCOS. The International Guidelines for the Assessment and Management of PCOS recommend lifestyle interventions such as diet and exercise as the first line of treatment [8]. Up to 70–80% of women with PCOS are overweight or obese and obesity is an evolutionary mismatch disorder that is a significant driver of the global rise in NCDs [36,37]. Effective intervention strategies require a realignment of lifestyle factors with ancestral living patterns—such as whole-food diets, regular physical activity, stress management, and circadian rhythm alignment [38]. This Special Issue features several papers that highlight the significant role of lifestyle factors in PCOS (contributions 4–6). Other options include pharmaceuticals (metformin, GLP-1, Tirzepatide, SGLT-2), nutraceuticals (vitamins, minerals), herbs (thymoquinone—contribution 1), and nature-derived compounds (flaxseed oil, isoflavones, anthocyanins) [39].

## 2. An Overview of Published Articles

The article by Ercan et al. (contribution 1) evaluated the metabolic, hormonal, and ovarian effects of thymoquinone (TMQ) compared to metformin in a letrozole-induced PCOS rodent model. TMQ is the main bioactive component of *Nigella sativa* (black seed) and is known to have antioxidant and anti-inflammatory properties, modulate androgen synthesis, and improve glucose and lipid metabolism. Both treatments were associated with statistically significant improvements in body weight, hormone parameters, ovarian morphology, lipid profile, and IR. TMQ resulted in a greater elevation of HDL-C and reduced cystic follicles, and the metformin-treated group showed better HOMA-IR and reduced androstenedione levels.

Although this novel study demonstrated comparable significant improvements in metabolic, hormonal, and ovarian parameters in both the TMQ and metformin-treated groups, the underlying mechanisms were not investigated. Future research should explore gene- and protein-expression changes in related metabolic (AMPK, PI3K/Akt), inflammatory (CRP, TNF-α, IL-6), and hormonal pathways. In addition, careful human studies are needed to determine dosages and confirm safety and efficacy.

The second research paper by Opydo-Szmaczek et al. (contribution 2) explored the diagnostic potential of salivary biomarkers in distinguishing adolescents with PCOS (n = 41; aged 15–19 years) from healthy controls (n = 31; aged 15–19 years). Standardized measurements were taken for salivary TNF-α, IL-6, IL-1β, testosterone, and uric acid, and a thorough dental examination was performed to assess oral hygiene and gingival status. PCOS participants had significantly higher BMI than controls (mean = 25.2 vs. 19.9 kg/m^2^) and there were no significant differences between gingival and plaque indices. Receiver operating characteristic (ROC) curve analysis was performed to evaluate the diagnostic performance of salivary biomarkers. Salivary testosterone was positively correlated with serum testosterone (Pearson’s r = 0.4664, *p* = 0.0021), and TNF-α, IL-6, and IL-1β showed high diagnostic accuracy (Area under the curve (AUC) = 0.921, 0.891, and 0.870, respectively). The high sensitivity and specificity for inflammatory biomarkers (TNF-α sensitivity 89.49%, specificity 80%; IL-1β sensitivity 89.47%, specificity 88%), supports the significance of inflammation in the pathophysiology of PCOS.

While salivary biomarkers offer a valuable non-invasive tool for the early diagnosis of PCOS in adolescents, it should be noted that this was a pilot study and had certain limitations. The cross-sectional design restricts conclusions regarding causality and temporal changes in biomarkers. The small sample size and absence of blood tests in controls limits between-group comparisons and generalizability. The study group consisted of a heterogenous mix of PCOS phenotypes that included a relatively small number of adolescents with hyperandrogenism. This pilot study lays the groundwork for future research focused on larger longitudinal cohorts in diverse phenotypes using direct comparisons between salivary and serum biomarkers.

An important and often under-recognized dimension of PCOS is the significant psychological burden carried by many women, a finding clearly demonstrated in the study by Marschalek et al. (contribution 3), which compared 31 women with PCOS with 31 healthy controls, using the validated SCL-90-item psychological symptom questionnaire. The authors showed that women with PCOS recorded significantly higher scores in seven of the nine SCL-90 domains, together with elevations across all three global indices of distress. These findings remained significant after adjustment for age and BMI. Parallel reductions were also observed across almost all subdomains of the Short Form-36, a widely used measure of health-related quality of life, which underscores the broader psychosocial impact of PCOS. Notably, psychological distress did not correlate with typical clinical or biochemical features of PCOS, which suggests that the emotional burden reflects a wider lived experience of the condition rather than a direct expression of hyperandrogenism, weight, or menstrual disturbances.

The study also identifies perceived stress and coping capacity as central influences on psychological symptoms. Higher distress was strongly associated with greater perceived stress and helplessness, and lower self-efficacy, which is consistent with wider literature showing that coping strategies shape both emotional wellbeing and quality of life in PCOS. The convergence between SCL-90 domains, perceived stress measures, and SF-36 outcomes supports the reliability of the SCL-90 in this population and reinforces the guideline recommendation to incorporate routine mental health assessment into clinical care. These findings highlight that emotional regulation, stress management, and supportive care pathways are essential components of comprehensive PCOS management rather than optional additions, and they align with the experiences reported by women living with PCOS.

Parker et al. (contribution 4) have proposed a comprehensive insulin-centric model for the assessment and management of IR as an alternative to the current glucose-focused paradigm. A major limitation of the prevailing glucose-centric approach is that measurable changes in serum glucose do not occur until decades after the onset of IR and hyperinsulinemia. As a result, IR frequently goes undetected during its early, silent phase, when intervention would be most effective. The implementation of an insulin-centric approach represents a paradigm shift that would enable timely intervention and reduce the risk of subsequent metabolic and reproductive complications.

Future research should involve development of consensus guidelines regarding the implementation of existing evidence-based surrogate markers for IR testing (e.g., serum insulin, HOMA-IR) coupled with an ongoing evaluation of real-world performance. Clinicians should be informed about the limitations of using surrogate markers and learn to interpret the results in relation to other tests, such as dynamic glucose–insulin testing, continuous glucose monitoring, anthropomorphic data, and inflammatory biomarkers, in the context of personalized history and examination. IR testing needs to be integrated with targeted lifestyle and medical interventions. The introduction of an insulin-centric model requires a coordinated international effort, as we have seen with the development of the International Guidelines for PCOS, and could be integrated into the existing global network.

Arvanitakis et al. have reviewed the complex interplay between the shared pathophysiological mechanisms of MASLD and PCOS (contribution 5). These include metabolic dysregulation, IR, hyperinsulinemia, dyslipidemia, lipotoxicity, chronic systemic inflammation, and obesity. Not surprisingly, women with PCOS have a high risk of developing MASLD and the authors recommend screening for MASLD in women with PCOS. Legacy terms like non-alcoholic fatty liver disease (NAFLD) have focused on end-stage manifestations, obscuring shared pathogenic origins. The current international consensus process to change the name of PCOS also recognizes the systemic metabolic nature of PCOS. This aligns with the proposal for the introduction of an insulin-centric model for the early assessment of metabolic disturbance in PCOS (contribution 4).

Elenezi et al. have performed a systematic review of diet-induced weight-loss on inflammatory status and hyperandrogenism (contribution 6). They included randomized controlled trials and cohort studies and identified 11 studies for systematic review and 9 for meta-analysis, according to PRISMA guidelines. Pooled analysis showed a statistically significant decrease in CRP (SMD 0.39, 95%CI 0.22, 0.56), IL-6 (SMD 0.37, 95%CI 0.12, 0.61), TNF-α (SMD 0.30, 95%CI 0.07, 0.53), androstenedione (SMD 0.36, 95%CI 0.13, 0.60), and LH (SMD 0.30, 95%CI 0.09, 0.51). The authors concluded that diet-induced weight-loss improves PCOS-related chronic inflammation and hyperandrogenism. This data further supports the recommendations of the International Guidelines and reinforces the pressing need to fast-track diet-related intervention strategies for the management of PCOS.

## 3. Future Research Related to Published Articles

A recent collaboration of the PCOS International Guideline Network generated a consensus roadmap for 150 clinical research priorities in PCOS [40]. Surveys of women, stakeholder groups, and healthcare providers called for an enhanced focus on the metabolic aspects, and a greater understanding of the long-term metabolic implications of PCOS. The Guideline group recognized the need for discovery science research related to the pathophysiology of PCOS, but noted that this was “outside the scope of this clinical research roadmap which focuses on enhancing current clinical practices and alignment with immediate clinical care needs for PCOS” [40]. The key areas identified include:

1. Optimizing PCOS diagnosis and preventing complications.
2. Develop evidence-based resources and explore optimal information provision.
3. Exploring effective lifestyle and weight management strategies.
4. Exploring intervention effects on diverse features of PCOS.
5. Optimizing preconception care and fertility treatments in PCOS.

The shift in emphasis from mechanistic research to translational research and implementation of existing preventative, diagnostic and therapeutic strategies should be a priority for governments, health administrators, women’s health advocates, and clinicians. The available research base—26,704 peer-reviewed papers on PCOS on PubMed (as of 2 December 2025)—provides substantial evidence to support immediate implementation of a number of high-priority practical strategies. These include:

1. Increased efforts to diagnose PCOS at an early stage in adolescence through *education and awareness programs* [41,42].
2. Increased *implementation of key areas of lifestyle* intervention (diet, exercise, sleep, stress, social support, management of obesity) using personalized and public health initiatives [43].
3. Early diagnosis of metabolic dysfunction with *biomedical testing for insulin resistance* (contribution 4).
4. *Preconception and antenatal counseling and lifestyle interventions*—combined with validated biomedical screening tools—to improve fertility and reduce pregnancy-related complications [44].
5. Promotion of *screening strategies to identify women at increased risk of cardiometabolic morbidity and mortality* due to previous pregnancy-related complications such as gestational diabetes, fetal growth restriction, and pre-eclampsia [45].

## 4. Conclusions

This compilation of articles explores a range of new challenges and perspectives in PCOS related to metabolism, immune function, progression to chronic disease, lifestyle, biomarkers, mental health, and pharmacological and natural treatment options. Many of the articles in this Special Issue directly address the translational focus of the International Guideline Network roadmap. Significant effort should now be directed at implementing existing evidence-based strategies to reduce the clinical impact of PCOS throughout the different life stages. Effective intervention in adolescents and adults has the potential to improve symptoms and quality of life, decrease morbidity and mortality, and reduce transgenerational transmission of PCOS.

## Data Availability

Not applicable.

## Abbreviations

The following abbreviations are used in this manuscript:

Akt	Protein kinase B
AI	Artificial intelligence
AMH	Antimullerian hormone
AMPK	Adenosine monophosphate-activated protein kinase
CRP	C-reactive protein
CSI	Chronic systemic inflammation
CVD	Cardiovascular disease
DHEAS	Dehydroepiandrosterone sulfate
FSH	Follicle-stimulating hormone
GLP-1	Glucagon-like peptide-1
HDL-C	High-density lipoprotein—cholesterol
HOMA-IR	Homeostatic model assessment for insulin resistance
HPA	Hypothalamic–pituitary–adrenal
IL	Interleukin
INRS	Insulin receptor
INS1/2	Insulin receptor substrate 1 and 2
IR	Insulin resistance
LH	Luteinizing hormone
MASLD	Metabolic-associated steatotic liver disease
NCD	Non-communicable disease
OGTT	Oral glucose tolerance test
PCOS	Polycystic ovary syndrome
PI3K	Phosphoinositide 3-kinase
PPAR-Ɣ	Peroxisome proliferator-activated receptor gamma
PRISMA	Preferred reporting items for systematic reviews and meta-analyses
ROC	Receiver operating characteristic
SGLT-2	Sodium-glucose co-transporter 2
SHBG	Sex hormone-binding globulin
T2DM	Type 2 diabetes mellitus
TMQ	Thymoquinone
TNF-α	Tumor necrosis factor-alpha
